# Effect of Physicians' Attitudes and Knowledge on the Quality of Antibiotic Prescription: A Cohort Study

**DOI:** 10.1371/journal.pone.0141820

**Published:** 2015-10-28

**Authors:** Cristian Gonzalez-Gonzalez, Paula López-Vázquez, Juan Manuel Vázquez-Lago, María Piñeiro-Lamas, Maria Teresa Herdeiro, Pilar Chávarri Arzamendi, Adolfo Figueiras, GREPHEPI Group

**Affiliations:** 1 Department of Preventive Medicine and Public Health, University of Santiago de Compostela, Santiago de Compostela, Spain; 2 Consortium for Biomedical Research in Epidemiology & Public Health (*CIBER en Epidemiología y Salud Pública—CIBERESP*), Santiago de Compostela, Spain; 3 Polytechnic and University Teaching Institute (*Cooperativa de Ensino Superior Politécnico e Universitário*—*CESPU*), Gandra PRD, Portugal; 4 Institute for Advanced Research and Training in Health Sciences and Technologies, Gandra PRD, Portugal; 5 Northern Pharmacovigilance Unit, Faculty of Medicine, University of Porto, Porto, Portugal; 6 Subdirectorate-General of Pharmacy, Galician Health Service (*Servicio Gallego de Salud*—*SERGAS*), Santiago de Compostela, Spain; Cairo University, EGYPT

## Abstract

Resistance increases with the use and abuse of antibiotics. Since physicians are primarily responsible for the decision to use antibiotics, ascertaining the attitudes and knowledge that underlie their prescribing habits is thus a prerequisite for improving prescription. Three-year follow-up cohort study (2008–2010) targeting primary-care physicians (n = 2100) in Galicia, a region in NW Spain. We used data obtained from a postal survey to assess knowledge and attitudes. A physician was deemed to have demonstrated Appropriate Quality Prescription of Antibiotics (dependent variable) in any case where half or more of the indicators proposed by the European Surveillance of Antimicrobial Consumption had values that were better than the reference values for Spain. The mail-questionnaire response rate was 68·0% (1428/2100). The adjusted increase in the interquartile OR of displaying good prescribing of antibiotics for each attitude was: 205% for fear ("When in doubt, it is better to ensure that a patient is cured of an infection by using a broad-spectrum antibiotic"; 95%CI: 125% to 321%); 119% for better knowledge ("Amoxicillin is useful for resolving most respiratory infections in primary care"; 95%CI: 67% to 193%); and 21% for complacency with patients' demands ("Antibiotics are often prescribed due to patients' demands"; 95%CI: 0% to 45%). Due to the fact that physicians' knowledge and attitudes are potentially modifiable, the implementation of purpose-designed educational interventions based on the attitudes identified may well serve to improve antibiotic prescription.

## Introduction

Antibiotic resistance is a growing public health threat worldwide, due to the related morbidity and mortality and costs that this generates.[1] The principal factor behind the increase in resistance at a population and individual level is antibiotic misuse and abuse.[2] In Spain, as in most countries, the use of antibiotics occurs mainly in primary care,[2] where over 80% are consumed.[3] The latest European Surveillance of Antimicrobial Consumption (ESAC) reports indicate that Spain is a country with high community-wide antibiotic consumption levels,[4] which would account for the high rates of antibiotic resistance.[5]

Aside from being role models for other health professionals and patients, physicians are primarily responsible for the decision to use antibiotics.[1] Accordingly, it would be of great interest to identify how physicians' knowledge of and beliefs about issues of antimicrobial use and resistance could enhance the effectiveness of interventions targeted at improving antimicrobial use.[6] Nevertheless, we were unable to find any study that analyzed the relationship between physician's knowledge and attitudes and their misprescription and/or overprescription of antibiotics in clinical practice.[7]

Hence, this study sought to assess: (1) knowledge and attitudes concerning the antibiotic prescribing habits of primary-care physicians; (2) the quality and quantity of physicians' antibiotic prescribing according to ESAC indicators; and, (3) the influence of physicians' knowledge and attitudes on the quality and quantity of antibiotic prescription.

## Methods

### Setting

Galicia is a region in NW Spain with a population of 2.8 million, 31% of whom are >65 years of age. Practically 100% of the population is covered by the Spanish National Health System (NHS). The NHS affords universal coverage, which is almost fully funded by taxes and is predominantly within the public sector. Provision of all health services, other than pharmaceutical, is free of charge at the point of delivery.

Prior to April 2012, pharmaceutical services were co-financed by outpatients, with pensioners and their beneficiaries being exempt from co-payment, and non-pensioners and their beneficiaries paying 40% of the retail price. Since April 2012, there has been a change in the payment for medication, in that patients' contributions are now income-linked.

In Spain, medication may only be dispensed by community pharmacies, and for some types of medication, e.g., antibiotics, a physician's prescription is compulsory; even so, the dispensing of drugs without a prescription continues to exist in Spain.[8]

### Study design, population and sample

We designed a cohort study covering NHS general practitioners in Galicia (3675 actively employed in 2010). The following were excluded: (1) residents undergoing training; (2) *locum tenens* and temporary staff, and (3) physicians who worked exclusively in emergencies. The questionnaire was sent to all primary-care physicians in Galicia who met the inclusion criteria, along with a letter of presentation outlining the study objectives.

### Ethics Statement

The study was approved by the Galician Ethics Committee (code number 2007/107). To ensure confidentiality, once the data needed to obtain the prescription indicators had been linked to the results of the questionnaires, they were furnished by the Galician NHS in an anonymized format such that no indicator could be related to a specific professional.

### Data-collection of exposure measures

Attitudes and knowledge concerning antibiotics and resistance were measured using a self-administered questionnaire mailed to the participants during the course of 2010.

The questionnaire was accompanied by: (1) a letter explaining the study objectives and the importance of participating; (2) a prepaid addressed envelope for returning the completed questionnaire, and (3) a small gift in the form of a ballpoint pen or pencil. All the materials were silk-screened with the logotype of the academic institution that sponsored the study. In the case of non-respondents, the questionnaire was resent a maximum of four times.

The questionnaire was designed on the basis of the results of a qualitative study conducted on the same population.[9] Physicians' knowledge of and attitudes to antibiotics and resistance were measured using a visual analog scale, which was scored from zero to ten according to their degree of agreement with the statement[10] The items included in the questionnaire were drawn from the results of the previous bibliographic review [7] and qualitative study.[9] These items assess *knowledge* of:

- the importance of resistance to antibiotics (Table 1, item 1);
- the usefulness of diagnostic tests in primary care (Table 1, item 2);
- the relationship between overprescribing and antibiotic resistance (Table 1, item3); and,
- the most useful antibiotics in primary care (Table 1, item 11).

and the following *attitudes*:

- *complacency*: attitude that motivates the prescribing of antibiotics to fulfill professionals' perceptions of their patients'/parents' expectations (Table 1, item 9),
- *fear*: attitude relating to fear of possible future complications in the patient (Table 1, items 5, 7, 8); and,
- *external responsibility*: attitude underlying the belief that responsibility for generating antibiotic resistance lies with other professionals (Table 1, items 4, 6, 10).

**Table 1:** Association between knowledge and attitudes, and antibiotic prescribing quality. OR of a physician displaying Appropriate Quality Prescription of Antibiotics (AQPA) versus not displaying AQPA, on comparing the 25^th^ with the 75^th^ percentile of each knowledge and attitude.

Knowledge and attitudes^a^	Percentile			IqOR comparing the 25^th^ with the 75^th^ percentile	
	25	50	75	IqOR (95% CI)	*p*-value
1. Antibiotic resistance is a major public health problem in our setting.^b^	7.5	9.5	9.5	0,94 (0.79–1.10)	0.424
2. In primary care it is useful to wait for a microbiology result when treating infectious diseases.^b^	1.5	3.0	5.5	1.22 (0.96–1.57)	0.099
3. The prescription of an antibiotic to a patient does not influence the development of resistance.^b^	1.0	1.5	4.0	0.75 (0.61–0.91)	0.005
4. New antibiotics will be developed to solve the problem of resistance.^c^	2.5	5.0	6.5	0.72 (0.55–0.92)	0.008
5. When in doubt, it is better to ensure that a patient is cured of an infection by using a broad-spectrum antibiotic.^e^	2.5	5.0	7.5	0.33 (0.24–0.44)	<0.001
6. The use of antibiotics in animals is a major cause of the occurrence of new resistance.^c^	4.5	6.5	8.5	1.52 (1.17–1.94)	0.002
7. Antibiotics are often prescribed because it is impossible to track the patient accurately.^e^	1.0	3.0	5.5	0.51 (0.39–0.65)	<0.001
8. When in doubt as to whether a patient has a bacterial disease, it is best to prescribe an antibiotic.^e^	1.5	3.5	5.5	0.60 (0.47–0.78)	<0.001
9. Antibiotics are often prescribed due to patients' demands.^d^	0.5	1.0	2.5	0.83 (0.69–1.00)	0.054
10. If patients believe that they need an antibiotic and the doctor does not prescribe it, they will get it at the pharmacy without a prescription.^c^	2.5	5.5	8.0	0.89 (0.63–1.18)	0.384
11. Amoxicillin is useful for resolving most respiratory infections in primary care.^b^	5.0	7.5	9.5	2.19 (1.67–2.93)	<0.001

Abbreviations: IqOR: Interquartile odds ratio; CI: confidence interval; AQPA = Half or more ESAC indicator values better than the reference values for Spain. [12]

^a^ Measured using a continuous, horizontal, visual analog scale. Recorded answers were scored in a range from zero (total disagreement) to ten (total agreement).

^b^ Knowledge.

^c^ External responsibility.

^d^ Complacency.

^e^ Fear

The linguistic aspects of the questionnaire were analyzed by experts, and the necessary amendments were then made to make it more readily understandable. Content and face validity were evaluated by a panel of experts, made up of specialists in clinical pharmacology, specialists in family and community medicine, pharmacists, specialists in epidemiology and preventive medicine, specialists in methodology, and psychologists. The reproducibility of the questionnaire was assessed by conducting a pilot study on 100 physicians.

### Follow-up and outcome measures

The designated follow-up period was 2008 through 2010. The Galician NHS provided monthly online prescription records (drug-indication information was unavailable) for physicians studied from January 2008 to December 2010.

Using the above data, we proceeded to make the calculations required to obtain the ESAC indicators in three steps:

- first, we calculated the overall number of DDDs (the assumed average maintenance dose per day for a drug used for its main indication in adults[11]) prescribed by each physician in each year for all his/her patients;
- second, based on the number of DDDs, and taking into account the number of persons allocated to each physician in each year of follow-up, we calculated antibiotic use expressed in DDDs per 1000 inhabitants per day (DID)[11]; and
- finally, we used these data to calculate the following prescription quality indicators proposed by the ESAC and previously validated by Coenen *et al*[12]:
  - J01_DID: consumption of antibacterials for systemic use (J01) expressed in DID;
  - J01C_DID: consumption of penicillins (J01C) expressed in DID;
  - J01D_DID: consumption of cephalosporins (J01D) expressed in DID;
  - J01F_DID: consumption of macrolides, lincosamides and streptogramins (J01F) expressed in DID;
  - J01M_DID: consumption of quinolones (J01M) expressed in DID;
  - J01CE_%: consumption of b-lactamase-sensitive penicillins (J01CE) expressed as a percentage;
  - J01CR_%: consumption of combinations of penicillins, including b-lactamase inhibitors (J01CR), expressed as a percentage;
  - J01DD+DE_%: consumption of third- and fourth-generation cephalosporins [J01(DD+DE)] expressed as a percentage;
  - J01MA_%: consumption of fluoroquinolones (J01MA) expressed as percentage;
  - J01_B/N: ratio of the consumption of broad-spectrum {J01[CR+DC+DD+(F-FA01)] to the consumption of narrow-spectrum penicillins, cephalosporins and macrolides [J01(CE+DB+FA01)]
  - J01_SV: seasonal variation in total antibiotic consumption (J01); and,
  - J01M_SV: seasonal variation in quinolone consumption (J01M).

These indicators were calculated per physician per year. The use of these indicators lent a number of strengths to our study: (1) their face validity and potential applicability have been deemed acceptable by experts from 15 European countries;[12] (2) their use ruled out any possible opportunistic selection of indicators for the purposes of our study; and, (3) they facilitated comparability with similar studies at a European level.[13]

A physician was deemed to have demonstrated Appropriate Quality Prescription of Antibiotics (AQPA) in any given year (dependent variable), where half (n = 6) or more of his/her ESAC indicator values were better than the reference values for Spain as a whole,[14]. Physicians having 6 or more indicators worse than the reference values for Spain as a whole were deemed not to have shown AQPA.

### Analysis

The questionnaire's reproducibility was evaluated using the intraclass correlation coefficient (ICC).

Generalized linear mixed models were subjected to statistical analysis. This statistical method allows for longitudinal data analysis (repeat and multiple observations over time on each of many individuals), with the advantage that application of these models for repeated measures affords greater statistical power than do ordinary regression models. Moreover, these models allow for the introduction of random terms to control initial intra-individual heterogeneity. To construct the models, we used AQPA as the dependent variable, with individual observations (per year and physician) as level 1 and physicians as level 2; random effects were considered among physicians.

In view of the fact that the response variable was dichotomous (displaying AQPA = 1 vs. not displaying AQPA = 0), these models were fitted with the binomial family, using the lme4 package for the R free software environment for statistical computing. [15] The results were expressed in ORs adjusted for active duty in on-call rotas and night shifts and the mean number of regular patients allocated to each physician. To take into account the independent variable's scale and its distribution among the study subjects, we calculated the interquartile OR (IqOR), which is based on an incremental exposure corresponding to the interquartile range of these attitude measures. Since most ORs assume values lower than unity, we calculated the inverse of the IqOR (1/IqOR), which can be interpreted as the increase in the probability of having AQPA when exposure decreases from the 75^th^ to the 25^th^ percentile of the distribution.

## Results

### Questionnaire development and reliability

The questionnaire was initially made up of 16 items but only 11 were considered for the final analysis. All of these had ICCs of over 0.5.

### Participants

**Fig 1:**
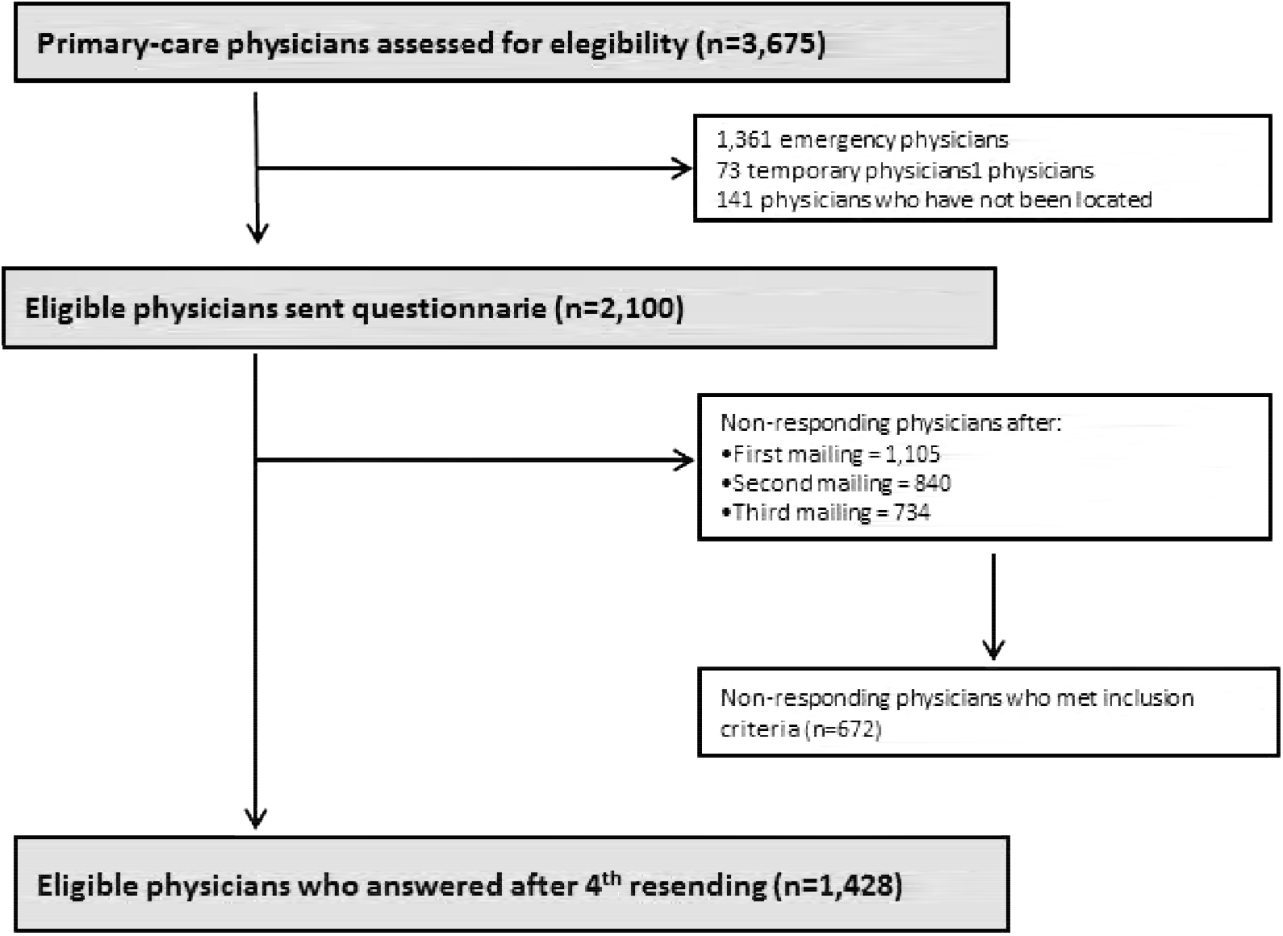
Flow of participants through the study.

Of the total population of primary-care physicians in Galicia (N = 3675), we selected those who, according to the records, fulfilled the inclusion criteria and could be located (n = 2100). Percentage response was 47.4% (n = 995) after the first mailing of the questionnaire, rising to 60.0% (n = 1260) after the second mailing and to 65.0% (n = 1366) after the third mailing. After the fourth and last mailing, 68.0% answered the questionnaire (n = 1428) (see Fig 1).

### Responders vs. non-responders

Table 2 shows the values of the ESAC indicators for all subjects in the sample, participants and non-participants alike. The indicator values for responders and non-responders were very similar.

**Table 2:** Percentage (PCT) of physicians having better values than the Spanish reference value for each European Surveillance of Antimicrobial Consumption (ESAC) indicator,[12] in both groups, i.e., questionnaire responders and non-responders.

Indicators [12]	Spanish reference values	Total		Responders		Non-responders	
		Value	(PCT> ref value) ^c^	Value	(PCT> ref value) ^c^	Value	(PCT> ref value) ^c^
1. DID of antibiotics for systemic use	19.68	15.65	(21.86)	15.59	(21.20)	15.71	(22.51)
2. DID of penicillins	12.31	8.66	(15.70)	8.81	(16.72)	8.50	(14.67)
3. DID of cephalosporins	1.56	1.92	(44.83)	1.89	(43.95)	1.94	(45.71)
4. DID of macrolides, lincosamides and streptogramins	1.90	1.73	(31.46)	1.65	(29.19)	1.80	(33.72)
5. DID of quinolones	2.42	2.15	(35.06)	2.05	(32.80)	2.24	(37.32)
6. % of beta-lactamase sensitive penicillins ^a^	0.50	0.43	(15.68)	0.54	(18.71)	0.31	(12.64)
7. % of combinations of penicillins with beta-lactamase inhibitors ^a^	38.70	41.75	(58.37)	41.90	(59.59)	41.60	(57.15)
8. % of third- and fourth-generation cephalosporins ^a^	2.80	4.34	(45.46)	4.38	(47.01)	4.30	(43.91)
9. % of quinolones ^a^	12.0	13.43	(60.93)	12.92	(58.16)	13.93	(63.70)
10. Ratio of the consumption of broad- to the consumption of narrow-spectrum penicillins, cephalosporins and macrolides	56.89	220.09	(86.12)	224.90	(86.63)	215.28	(85.61)
11. Seasonal variation in consumption of antibiotics ^b^	125.8	23.18	(3.96)	24.24	(3.21)	22.11	(4.71)
12. Seasonal variation in consumption of quinolones ^b^	117.3	26.54	(13.52)	26.61	(14.71)	26.46	(12.32)

Abbreviations. PCT: percentage DID: doses inhabitant day

^a^ Percentage of total consumption of antibacterials for systemic use (J01) in DID.

^b^ Overuse in winter quarterly periods (October–December and January–March) compared with the summer quarterly periods (July–September and April–June) over a 1-year period starting in July of one calendar year and ending in June of the following year, expressed as a percentage: [DDD (winter quarterly periods)/DDD (summer quarterly periods)-1]×100.

^c^ Percentage of physicians with values above the reference value.

### Study vs. Spanish quality indicators

Table 2 also shows the values of each of the ESAC indicators for Spain. The physicians who participated in the study registered higher values for indicator 3 (defined daily dose per 1000 inhabitants per day (DID) of cephalosporins), indicator 7 (percentage of combinations of penicillins with beta-lactamase inhibitors), indicator 8 (percentage of third- and fourth-generation cephalosporins), indicator 9 (percentage of quinolones), and indicator 10 (ratio of the consumption of broad- to the consumption of narrow-spectrum penicillins, cephalosporins and macrolides). In addition, the study subjects showed a lower percentage consumption of beta-lactamase-sensitive penicillins (indicator 6).

### Association between knowledge and attitudes, and prescription indicators

Table 1 shows the result of the regression to quantify the association between the degree of agreement with the 11 items (related with knowledge and attitudes) and the fact of having demonstrated AQPA. The *fear*-related items showed a greater effect magnitude, with 1/IqORs of: (i) 205% for "When in doubt, it is better to ensure that a patient is cured of an infection using a broad-spectrum antibiotic" (95%CI: 125% to 321%); (ii) 97% for "Antibiotics are often prescribed because it is impossible to track the patient accurately" (95%CI: 53% to 158%); and (iii) 67% for "When in doubt whether the patient has a bacterial disease, it is better to prescribe an antibiotic" (95CI: 28% to 111%).

*Knowledge* was also associated with having AQPA, with: (i) an IqOR of 119% for "Amoxicillin is useful for resolving most respiratory infections in primary care" (95%CI: 67% to 193%); (ii) an 1/IqOR of 33% for "The prescription of an antibiotic to a patient does not influence the development of resistance" (95%CI: 10% to 63%); and (iii) an 1/IqOR of 40% for "New antibiotics will be developed to solve the problem of resistance (95%CI: 8% to 83%).

Finally, *complacency* was likewise associated with AQPA, with an 1/IqOR of 21% for "Antibiotics are often prescribed due to patients demands", though this was at the limit of statistical significance: 95%CI: 0% to 45%, *p* = 0.054.

## Discussion

This large-scale retrospective cohort study is the first to show that physicians' attitudes and knowledge determine the quality of prescription of antibiotics, as measured by indicators obtained from clinical practice. Our results indicate that fear of complications, complacency with patients, and insufficient knowledge are the factors related with the prescribing of antibiotics by general practitioners. In view of the fact that knowledge and attitudes are potentially modifiable, this study supports the contention that implementation of strategies specifically targeted at changing these factors could reduce misprescription of antibiotics in primary care.

The *mean consumption* of antibiotics in primary care in NW Spain was 15.65 DID. Bearing in mind that 85% of all prescriptions in Spain are generated in primary care, this would indicate that consumption in total DID would be around 18.41, a figure very similar to the Spanish mean for 2009 (19.68).[16] Nevertheless, it will be seen from our study that the indicators which assess the ratio of broad- to narrow-spectrum antibiotics are worse than the indicators for Spain as a whole, and worse than those for most European countries. These results could be of great interest when it comes to designing interventions aimed at improving prescription.

There are many *qualitative* studies that explore physicians' subjective perceptions of the factors, attitudes and knowledge which influence antibiotic prescribing. Due to their nature, however, such studies are unable to determine which of these are associated with inappropriate prescription of antibiotics in clinical practice, or the magnitude of their association. A recent review of qualitative studies found that the attitudes most frequently cited by physicians as influencing their prescribing were *complacency* and *fear*, [17]a finding in line with the results of our study.

Previous *quantitative* studies which linked physicians' attitudes to their prescription of antibiotics are inconsistent. Most of these studies used fictitious cases posed in a questionnaire to simulate prescribing, and then linked this to attitudes measured in the same questionnaire.[7] By using simulated prescription, no factor other than knowledge could ever be detected. Few studies used questionnaires and reviews of clinical histories or prescriptions to link attitudes to prescribing in real clinical practice. Moreover, none of them made a general assessment of how knowledge or attitudes can influence the prescription of antibiotics.[7] Despite the methodologic limitations of these studies, it seems that *complacency* and, to a lesser extent, *fear*, emerge as possible factors which affect the misprescription of antibiotics.

The finding that *fear* is the principal attitude associated with the quality of prescription is internally consistent with the marked trend of the physicians in our study to prescribe broad-spectrum antibiotics. Consequently, the prescription quality indicators that relate the use of broad- to narrow-spectrum antibiotics are those which diverge most from the Spanish values. Fear may also be responsible for a greater prescription of antibiotics for processes for which they are not indicated. The effect exerted by fear on antibiotic prescribing could be counteracted by (1) delayed prescribing, (2) physician training, and (3) the use of clinical decision support systems (CDSS). *Delayed prescribing* is a good strategy for reducing the quantity of antibiotics, while maintaining clinical outcomes.[18] Furthermore, CDSS and the continuing education of health professionals can also serve to reduce fear (by providing support to increase physicians' certainty) and its consequences.

Physicians' *complacency* with patients is another of the factors that have been repeatedly proposed as determinants of prescription. We observed that complacency was associated with prescription, though to a lesser degree than fear. Physicians' perceptions of patients' expectations are often translated as misprescription of antibiotics, due to their efforts to maintain a good physician-patient relationship.[19] Complacency may thus be linked to: (1) patients' attitude to antibiotics, especially in Spain where patients are more prone to use antibiotics, even for treating viral illnesses,[20]; and (2) the physician-patient relationship and mean time per consultation (the shorter the consultation time, the fewer possibilities of the patient becoming involved in decision-making).[21] Interventions targeted at enhancing physicians' communicative capacities (non-verbal communication), [22] better managing their mean time per consultation, and promoting delayed prescribing could improve the influence of complacency on prescription. Such techniques would make patients feel that they were being attended by the physician and involve them in the decision-making process.[23] Indeed, recent studies show that most patients are satisfied with delayed prescribing.[24]

*Insufficient knowledge* is a factor inversely associated with AQPA. One of the attitudes that best reflects this characteristic is, "Amoxicillin is useful for resolving most respiratory infections in primary care". Indeed, the highest level of knowledge can be associated with AQPA, and is reflected in the increase in total DID and the higher proportion of broad-spectrum antibiotics. The principal strategies for improving insufficient knowledge might lie in continuing education and training for professionals, and the use of CDSS.

### Strengths

Our study possesses a series of strengths: *firstly*, outcomes were generated, not from fictitious prescriptions, but rather from real data sourced from clinical practice. The use of real data meant that the influence of fear or complacency could be assessed in the real scenario, something that cannot be done using fictitious prescriptions, which would only be capable of detecting a lack of knowledge.[7]

The *second* strength lay in the design of the questionnaire. This was developed on the basis of a systematic review conducted by our group and used to construct a theoretical model, which was in turn used to draw up an agenda for a qualitative study.[7] The qualitative study then identified the study populations' attitudes that had to be included by us in the questionnaire.[7,9] In addition, the questionnaire was rigorously validated in terms of reproducibility, face validity and content validity.[25]

The *third* strength was the high percentage participation achieved by the postal questionnaire (68%). [26] This reduced the risk of non-participation bias. Moreover, we were able to establish that there were no differences in indicators between the physicians who did and those who did not respond (Table 2). This further reduced the risk of non-participation bias. [27]

Among our study's advantages was its use of a visual analog scale, an instrument that has shown itself to be highly sensitive to change, as compared to the other methods of measuring degree of agreement in knowledge and attitudes, such as the Likert scale.[28,29] Moreover, the use of this type of scale means that the effect of the distribution of the answers in the study sample can be included in the expression of results, and so make it possible to analyze the IqOR and assess the effect of the change between the 25^th^ and 75^th^ percentiles, which is close to the potential for change in this attitude in the study population.

### Limitations

While lacking the indications for which the antibiotics were prescribed could be a limitation,[30] one can nevertheless assume that the populations attended by the physicians in the study (median of 1329 patients) would not present with important differences in the frequency of the principal diseases and/or indications for antibiotics. Accordingly, comparison of indicators could serve as a good approximation of prescription quality.

By way of indicators, we used those drawn up by the ESAC at a European level. [13] This not only enhanced our study's comparability, but also ruled out the possibility of opportunistic selection of indicators. To establish the cut-point between physicians with and without AQPA, we chose Spanish values as reference, since Spain possesses the characteristics (resistance patterns, clinical guidelines) that most closely resemble those of our study population. [12]

Another possible study limitation was the difficulty of assessing the validity criterion for attitudes, since there is no alternative reference method for measuring such attitudes. Yet, the fact that attitudes are capable of discriminating between physicians with and without AQPA would support the construct validity of these statements.

## Conclusions

Acute concern about resistance has indeed led to different interventions being implemented to improve the use of antibiotics worldwide. Rather than being introduced intuitively, however, such interventions should be based on gaps in knowledge, inappropriate attitudes, and mistaken beliefs detected in the target population.[31] The results of this study could thus be of great use in designing new interventions focused on modifying those attitudes that have been linked by us to misprescription.

## Abbreviations

AQPA: Appropriate Quality Prescription of Antibiotics
CDSS: Clinical Decision Support Systems
DID: Doses Inhabitant Day
ESAC: European Surveillance of Antimicrobial Consumption
IqOR: Interquartile OR
NHS: National Health System
